# Whole-genome resequencing data of the Ixworth chicken breed

**DOI:** 10.1186/s13104-026-07837-z

**Published:** 2026-05-04

**Authors:** Hendrik Bertram, Muhammad Jawad, Susann Michanski, Inga Tiemann, Armin O. Schmitt, Mehmet Gültas

**Affiliations:** 1 Statistics and Data Science Group, Department of Agriculture, South Westphalia University of Applied Sciences, 59494 Soest, Germany; 2 Breeding Informatics Group, Department of Animal Sciences, Georg-August University, 37075 Göttingen, Germany; 3 Precision Livestock Farming, Faculty of Agricultural Sciences and Landscape Architecture, Osnabrück University of Applied Sciences, 49076 Osnabrück, Germany; 4 Center for Integrated Breeding Research (CiBreed), Georg-August University, 37075 Göttingen, Germany

**Keywords:** Whole-genome re-sequencing, Chicken, Ixworth, Dual-purpose, Endangered, Local, Ecological farming, First public dataset

## Abstract

**Objectives:**

The Ixworth chicken is a British dual-purpose breed and mostly maintained by small-scale farmers. Due to legislation regarding the ban on male chick culling in European countries, such as in Germany, renewed interest has arisen in rearing dual-purpose chickens that provide both meat and eggs from the same genetic line. This dataset was generated within the scope of projects aiming to evaluate the viability of dual-purpose breeds for sustainable and welfare-oriented poultry production. One of the objectives was to characterize the genetic potential of the Ixworth chicken as a model for breeding programs that combine conservation and practical use in ecological farming systems.

**Data description:**

Liver samples from 49 male Ixworth chickens were collected after scheduled slaughter at the Campus Frankenforst of the Faculty of Agricultural, Nutritional and Engineering Sciences of the University of Bonn, Germany. Genomic DNA was extracted and subjected to whole-genome resequencing using the Illumina NovaSeq 6000 platform. The dataset provides high-resolution genomic information on a rare breed with a pure dual-purpose background. This resource represents the first public sequencing dataset of the Ixworth chicken and thus offers a valuable foundation for future studies on genetic diversity, conservation genomics, and breeding strategies for sustainable poultry production.

## Objective

Initiatives in recent years by the Federal Office for Agriculture and Food of Germany and the Ministry of Agriculture and Consumer Protection of North Rhine-Westphalia have funded several projects, including RegioHuhn [1], ÖkoGen [2], and Huhn3 [3], to promote the conservation and practical integration of local chicken breeds into more sustainable poultry farming. These efforts address both biodiversity preservation and the development of alternatives to the culling of male layer chicks, banned in Germany since 2022 [4]. Dual-purpose chickens, which provide both eggs and meat from the same genetic line, have emerged as a promising alternative, though their productivity generally lags behind specialized breeds [5–8].

Within a follow-up project to these initiatives, we collected genomic data from the Ixworth chicken, a local British dual-purpose breed mostly maintained by small-scale farmers from Great Britain [8, 9]. This dataset was generated to explore the breed’s genetic background, evaluate its suitability for sustainable dual-purpose production, and support conservation, as demonstrated by a previous study [9]. Therefore, a key objective was to provide the first publicly available whole-genome (re-) sequencing (WGS) dataset for this breed.

The presented WGS data enable assessment of genetic population parameters like genetic diversity, inbreeding, and selection signatures within the Ixworth population. The data complement prior work on dual-purpose breeds and provide a genomic foundation for future studies on breed conservation, genetic improvement, and comparative genomics in sustainable poultry systems.

## Data description

This dataset comprises WGS data from 49 male Ixworth chickens, a rare British dual-purpose breed. All individuals originate from a closed population of slightly more than 1,000 birds maintained at the Campus Frankenforst of the Faculty of Agricultural, Nutritional and Engineering Sciences, University of Bonn, Germany. In addition to this population, approximately 20 breeders in Great Britain [10] maintain an estimated number of 500 Ixworth chickens. The study population has been managed over more than six generations, with each generation producing ~ 150–170 chicks, from which 70 hens and 10 roosters are selected for breeding. Mating is random on the sire side, while eggs are individually recorded using trap nests, ensuring maternal traceability and resulting in low to moderate selection intensity. A detailed description of this population and its comparison to other dual-purpose and hybrid breeds is provided by Becker et al. [8]. Phenotypically, Ixworth cockerels reach ~ 2.34 kg at 12 weeks, with average daily gains of 27.4 g and a feed conversion ratio of 2.48 on a diet formulated for male layer lines. Carcass composition comprises 17.5% breast, 12.5% wing, and 31.8% leg yield. Hens produce about 195 eggs per laying cycle with an average egg weight of 56 g, initiate laying at 20 weeks, and reach peak laying performance of 70–80%. By 72 weeks of age, hens attain body weights exceeding 3 kg. The breed exhibits favorable robustness traits, including low mortality (1% during rearing and up to 5% during laying), high nest acceptance (> 97%), and good adaptation to free-range systems, with few observations of behavioral abnormalities or disease. Substantial inter-individual variation in growth and egg production indicates the presence of considerable segregating genetic diversity within the population. Hence, these circumstances are promising to further improve the performance of the Ixworth breed.

Liver tissues were obtained from 50 male Ixworth chickens immediately after routine slaughter. Samples were snap-frozen in liquid nitrogen and stored at −80 °C until DNA extraction. Approximately 20 mg of each liver sample was used for genomic DNA extraction with the peqGOLD Blood and Tissue DNA Mini Kit (VWR, Germany) according to the manufacturer’s protocol. Subsequently, RNase A digestion was included to remove RNA contaminants. Afterwards, DNA quality as well as concentration were assessed using a NanoDrop 2000 spectrophotometer (Thermo Fisher Scientific, USA) by measuring the 260/280 nm absorbance ratio. After normalization, DNA samples were adjusted to 50 µL. One sample was excluded after quality control, leaving 49 samples for sequencing.

Library preparation and WGS were performed by the Competence Centre for Genomic Analysis (Kiel, Germany) on an Illumina NovaSeq 6000 platform, producing 2 × 151 bp paired-end reads. The sequencing aimed for an average depth of 26x, yielding approximately 182.8 million reads per sample. The raw WGS reads (FASTQ files) have been deposited in the European Nucleotide Archive (ENA) under accession ERP172200, as described in Table 1.

**Table 1:** Overview of the WGS dataset of the Ixworth chicken breed

Label	Name of dataset	File types	Data repository and identifier
*Dataset 1*	*Whole-genome resequencing of the Ixworth chicken*	*FASTQ (.fastq.gz)*	*ENA Study ERP172200* (http://identifiers.org/ena.embl:ERP172200) [11]

## Limitations

Sampling was restricted to a single Ixworth chicken population maintained at one location, which may limit generalization to the wider breed. However, due to the critical status of the breed, it is likely not possible to obtain large numbers of samples from multiple populations. In addition, samples were obtained opportunistically from available carcasses after slaughter rather than by random selection, resulting in the inclusion of only male chickens. Hence, the sampling design may have led to a potential over-representation of related individuals within the dataset. Furthermore, pedigree records were unavailable and thus kinship can only be inferred from the genomic data. The mentioned factors may introduce bias in estimates of genetic diversity and other population-level parameters like effective population size.

## Data Availability

The data described in this Data note can be freely and openly accessed on ENA under study accession ERP172200 (http://identifiers.org/ena.embl:ERP172200). Please see Table 1 and reference [11] for details and links to the data.

## Abbreviations

WGS: Whole-genome (re-) sequencing
ENA: European Nucleotide Archive
